# Synaptic plasticity in schizophrenia pathophysiology

**DOI:** 10.1016/j.ibneur.2023.01.008

**Published:** 2023-02-04

**Authors:** Kexuan Zhang, Panlin Liao, Jin Wen, Zhonghua Hu

**Affiliations:** a Hunan Key Laboratory of Molecular Precision Medicine, Department of Critical Care Medicine, Xiangya Hospital, Central South University, Changsha 410008, Hunan, PR China; b Center for Medical Genetics, School of Life Sciences, Central South University, Changsha 410008, Hunan, PR China; c National Clinical Research Center for Geriatric Diseases, Xiangya Hospital, Central South University, Changsha 410008, Hunan, PR China; d Hunan Provincial Clinical Research Center for Critical Care Medicine, Xiangya Hospital, Central South University, Changsha 410008, Hunan, PR China; e Hunan Key Laboratory of Animal Models for Human Diseases, School of Life Sciences, Central South University, Changsha 410008, Hunan, PR China; f Key Laboratory of Hunan Province in Neurodegenerative Disorders, Central South University, Changsha 410008, Hunan, PR China

**Keywords:** Synaptic plasticity, Neurotransmission, Schizophrenia, Neuropsychiatric disease

## Abstract

Schizophrenia is a severe neuropsychiatric syndrome with psychotic behavioral abnormalities and marked cognitive deficits. It is widely accepted that genetic and environmental factors contribute to the onset of schizophrenia. However, the etiology and pathology of the disease remain largely unexplored. Recently, the synaptopathology and the dysregulated synaptic plasticity and function have emerging as intriguing and prominent biological mechanisms of schizophrenia pathogenesis. Synaptic plasticity is the ability of neurons to change the strength of their connections in response to internal or external stimuli, which is essential for brain development and function, learning and memory, and vast majority of behavior responses relevant to psychiatric diseases including schizophrenia. Here, we reviewed molecular and cellular mechanisms of the multiple forms synaptic plasticity, and the functional regulations of schizophrenia-risk factors including disease susceptible genes and environmental alterations on synaptic plasticity and animal behavior. Recent genome-wide association studies have provided fruitful findings of hundreds of risk gene variances associated with schizophrenia, thus further clarifying the role of these disease-risk genes in synaptic transmission and plasticity will be beneficial to advance our understanding of schizophrenia pathology, as well as the molecular mechanism of synaptic plasticity.

## 1 Introduction

Neurons are the structural and functional units of the central nervous system, which connect and communicate with each other through synapse, a intercellular junction transferring information from presynaptic neurons to postsynaptic cells (Südhof, 2018). This kind of connection is not invariable, but can be modulated, which called synaptic plasticity. Synaptic plasticity describes the morphological and functional changes in preexisting synapses of the nervous system in response to internal and external environmental stimuli (Citri and Malenka, 2008, Cramer et al., 2011). These activity-dependent modifications are essential for brain development, neuronal circuits establishment and synaptic transmission. Dysregulated synaptic plasticity has been involved in pathophysiology of numerous neuropsychiatric disorders (Stampanoni Bassi et al., 2019), such as schizophrenia, bipolar disorder, and major depressive disorder.

Schizophrenia is a severe psychiatric disorder affecting basic brain processes, such as thinking, perception and emotion (Kahn et al., 2015, Marder and Cannon, 2019). Patients with schizophrenia suffer from positive symptoms (hallucination, delusion, confused thoughts, disorganized speech, trouble of concentration), negative symptoms (anhedonia, alogia, social withdrawal, blunted affect, avolition), and abnormal cognition (Birnbaum and Weinberger, 2017). Typically, symptoms emerge at adolescence or early adulthood.

Researches on schizophrenic pathology mechanisms have been focused on brain connectivity alteration, anatomically manifested as structural changes of nerve fibers, functionally manifested as synaptic plasticity dysregulation. It is acknowledged that synaptic plasticity is involved in the onset of schizophrenia (Stephan et al., 2006), but the neuropathophysiology is not quite clear yet. In this review, we focus on the relationship between synaptic plasticity and pathology of schizophrenia. We hope to unravel the neurobiological basis of schizophrenia by summarizing multiple forms and mechanisms of synaptic plasticity, and schizophrenia animal models with deficient synaptic function and plasticity.

## 2 Etiology of schizophrenia

Genetics and epidemiological investigations have confirmed that both genetic and environmental factors contribute to schizophrenia etiology. Twin studies of schizophrenia suggest that genetic factors account for about 85% of schizophrenia (Tsuang, 2000). However, schizophrenia is not caused by a single risk gene (Trubetskoy et al., 2022), and it does not show a simple pattern of inheritance. Each genetic variances result in only a small increase in risk. Until the threshold level is reached and clinical symptoms appear. Moreover, environmental influences during prenatal and postnatal brain development or across adulthood, such as uterine infection or pregnancy complications, psychosocial causes, amphetamine abuse, autoimmune disease and other brain trauma, also affect the risk of schizophrenia.

## 3 Synaptic plasticity deficit is common pathological mechanism in schizophrenia

Heterogenous and complexity of schizophrenia pathology poses a great challenging to diagnosis and therapeutic intervention of the disorder. Unlike Alzheimer's and Parkinson’s disease that display pathological inclusion and neuron loss, schizophrenia lacks notable characteristic and hallmark in pathological change. Imaging studies (CT and Magnetic resonance examination) revealed the decrease in neocortical and hippocampal volumes, as well as enlarged ventricles in patients with schizophrenia (Harrison, 1999, Woods et al., 1996). Although structural abnormalities such as neuron loss in several brain regions have been revealed by postmortem studies, the results are fairly in consistence. For example, cortical loss is at least partly due to a loss of nerve fibers, including synapses, rather than neuron loss (Harrison, 1999).

Thus far, several lines of evidence have suggested that synaptic deficits including abnormal synaptic transmission and plasticity might be essential neuropathological feature of schizophrenia. Earlier neurochemical and recent genetic findings have implicated that glutamate, dopamine and GABA neurotransmitter dysfunction underlies the neurobiological basis of schizophrenia (Birnbaum and Weinberger, 2017), thereby leading to the hypothesis that excess or insufficient synaptic transmission might be related to pathology of the illness. Neuro-anatomical analysis of postmortem brain also shows that density of dendritic spines that are essential postsynaptic structure of neurons, is lower in cortical tissues from schizophrenia patients than that in controls, especially in layer 3 of the neocortex (Glausier and Lewis, 2013). Despite synapse loss, molecular alterations related to disturbance in synaptic function and plasticity have been implicated in schizophrenia. Indeed, large-scale gene expression examination indicates that presynaptic protein synaptophysin levels are decreased in the hippocampus, frontal cortex, and cingulate cortex in schizophrenia patients, while synaptic protein SNAP − 25, synapsin, rab3A, and PSD-95 are also reduced in the hippocampus in schizophrenia patients (Osimo et al., 2019). Therefore, understanding the disease-associated synaptic deficiency and the underlying mechanism are critical to elucidate the pathogenesis of schizophrenia and to develop new therapeutic interventions for the illness.

## 4 Molecular and cellular mechanism of synaptic plasticity

Synaptic plasticity, one of the most important properties of brain, refers to the ability of neurons to modify their connections in response to extrinsic stimuli. Neurons connect and exchange information through synapses, which are consist of presynaptic terminal, postsynaptic specialization or dendrite or cell (neuron) and a synaptic cleft between them. From birth to adulthood, the development of the brain involves the activity-dependent refinement of synapses and the construction of synaptic circuitry (Forrest et al., 2018). These modifications of the strength or efficacy of synaptic transmission during development and aging play central roles in brain functions such as thinking, perception, learning and memory, and have been widely recognized as a fundamental mechanism by which information is encoded and stored in the brain. Synaptic plasticity includes Hebbian plasticity that describes co-activity between neuron networks and homeostatic plasticity that acts as a compensative process to counterbalance the alteration of neuron network (Andersen et al., 2017, Zenke et al., 2017).

### 4.1 Short-term synaptic plasticity

Short-term synaptic plasticity lasts from milliseconds to minutes, which is important for the formation of short-term memory and transient behavioral response to environment (Citri and Malenka, 2008, Zucker and Regehr, 2002). Paired-pulse facilitation and depression, that are induced by delivery of two stimuli within a short interval, as well as post-tetanic potentiation (PTP) and depression, that are trigged by trains of stimulation, are several major forms of short-term synaptic plasticity. The key event for induction of facilitation in most forms of short-term plasticity is the rapid increase of presynaptic calcium level, which alters the probability of neurotransmitter release (Citri and Malenka, 2008). Transient depletion of readily releasable pool of synaptic vesicles or inactivation of voltage-dependent sodium or calcium channels might contribute to the depression at many synapses. In addition, desensitization of post-synaptic ligand-gated receptors and glia-neuron interaction are also involved in the mechanism of some forms of short-term synaptic plasticity (Araque et al., 1998, Haydon, 2001, Otis et al., 1996, Trussell et al., 1993).

### 4.2 Long-term synaptic plasticity

Long-term potentiation (LTP) and long-term depression (LTD) are two forms of Hebbian plasticity that biologically correlates for Hebb’s algorithm about network connection and contributes to structural and functional plasticity. In contrast to short-term synaptic plasticity, these two types of synaptic plasticity can last for hours or even days, and are recognized as essential mechanism for long-term memory storage.

LTP is the persistent increase of synaptic strength between neurons (Bliss and Gardner-Medwin, 1973). The most extensively studied forms of synaptic plasticity is NMDAR-dependent LTP (NMDAR-LTP) that can be induced by various protocols, such as high-frequency tetanic stimulation or ‘pairing protocol’ in which postsynaptic depolarization is coupled with low-frequency synaptic activation to stimulate pre-synaptic neurons, increasing the efficiency of synaptic transmission and excitability of post-synaptic neurons (Abraham and Huggett, 1997, Bliss and Lomo, 1973). LTP induction leads to calcium influx through NMDAR and an increase of calcium in dendritic spines, thereby activating intracellular signaling pathways and enrichment of AMPARs into the postsynaptic membrane surface (Diering and Huganir, 2018), which is vital for long-term learning (Lu et al., 2017, Malleret et al., 2010, Penn et al., 2017), memory consolidation and cognitive processes (Machado et al., 2018, Ren et al., 2020, Zhang et al., 2018). The activation and accompanied phosphorylation of calcium/calmodulin-dependent protein kinase II (CaMKII) are key molecule processes mediating post-synaptic mechanism of NMDAR-LTP. Firstly, phosphorylated CaMKII (pCaMKII) activates protein kinase A to stimulate the new synthesis of AMPARs through the cAMP-response-element-binding (CREB) protein pathway (Barco et al., 2002, Lisman et al., 2018, Wu et al., 2007). Secondly, pCaMKII phosphorylates postsynaptic scaffold protein and enhances endophilin A1 binding to the membrane, promoting the polymerization of actin to initiate the expansion of dendritic spines (Yang et al., 2021), which establish harbors for AMPARs. Thirdly, pCaMKII catalyzes the phosphorylation of AMPAR at residue Ser^831^ and Ser^845^ of the GluA1 subunit, increases its conductance and single channel open–probability, promotes AMPAR to locate to membrane surface (Lisman and Raghavachari, 2015, Lisman et al., 2012, Sanhueza et al., 2011). LTP is considered to be strongly facilitated with learning behavior, while is suppressed when exposed to a hazardous environment associated with impaired learning and memory performance (Jorgenson et al., 2005, Xiao et al., 2016, Zhang et al., 2021).

**Fig. 1:**
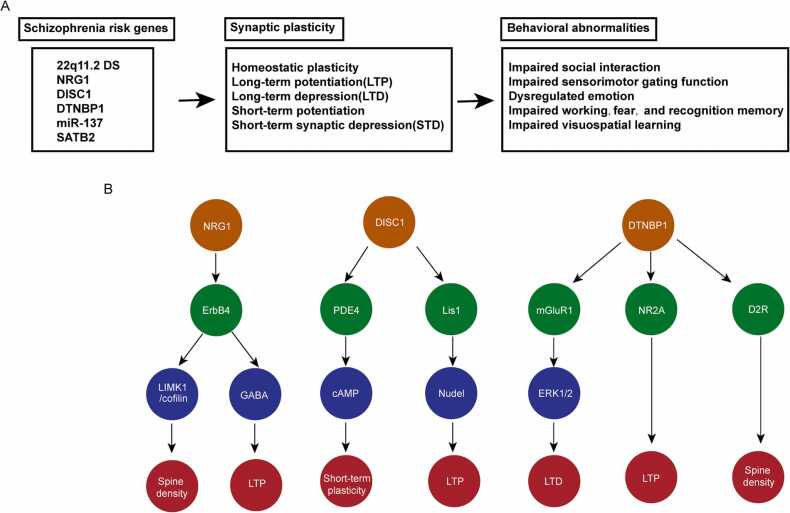
Mechanisms of synaptic plasticity in schizophreniaA. Summary of the role and mechanism of synaptic plasticity in schizophrenia pathology.B. A schematic illustration for the function of selected schizophrenia risk genes.

LTD is an prolonged decrease of synaptic transmission between neurons which can be triggered by repetitive low-frequency stimulations (LFS) (Mulkey and Malenka, 1992). NMDAR-dependent LTD induced at hippocampal Schaffer collateral-CA1 synapses is a major form of LTD that has been studied most extensively. The canonical mechanism of NMDAR-LTD induction in hippocampus involves calcium influx, activation of NMDAR, and the subsequent activation of a calcium-dependent protein phosphatase cascade. Moderate Ca^2+^ enters through NMDAR to activate CaM-dependent serine/threonine phosphatase calcineurin (CaN, also known as PP2B), which rescinds the inhibition to inhibitor-1 and in turn activates protein phosphatase 1 (PP1) (Collingridge et al., 2010, Mulkey et al., 1993). PP1 dephosphorylates various substrates crucial for expression of NMDAR-LTD, such as Ser^845^ of the GluA1 subunit and Ser^295^ of postsynaptic density protein 95 (PSD95) (Collingridge et al., 2010, Kim et al., 2007, Lee et al., 2000). Contrary to LTP, the main mechanism of LTD is CaN-mediated removal and endocytosis of AMPAR (Diering and Huganir, 2018). In addition, researches prove that transient recruitment and removal of Ca^2+^-permeable AMPA receptors mediated by AKAP150, PKA and CaN are necessary to LTD (Sanderson et al., 2016). LTD is known as a mechanism mediating synapse weakening in order to reconstruct useful synapse connection for LTP then to restore new information (Malleret et al., 2010, Stanton, 1996). LTD in hippocampus has been linked to cognitive process (Collingridge et al., 2010, Durkee et al., 2021, Navarrete et al., 2019). Compared with LTP, there are lots of blind spots to be figured out about how LTD regulate learning and memory. Fig. 1.

### 4.3 Homeostatic plasticity

Homeostatic plasticity refers to that neuron has the ability to maintain an appropriate excitability to adapt to sustained alteration of network activity (Surmeier and Foehring, 2004). Scaling up and scaling down are two forms of homeostatic plasticity, which are thought to counterbalance LTP and LTD in a negative feedback loop. Activity-dependent accumulation or dispense of AMPA receptors has been recognized as important mechanism of postsynaptic expression of homeostatic plasticity. Scaling up increases AMPARs expression and location while scaling down reduces AMPARs on the membrane (Diering and Huganir, 2018), both of which rely on PSD95- and MAGUKs (membrane-associated guanylate kinases)-mediated protein-protein interaction (Sun and Turrigiano, 2011) and phosphorylation of synaptic proteins that involved in cytoskeleton remodeling (Desch et al., 2021, Diering et al., 2017, Martin et al., 2019). Table. 1.

**Table 1:** Summary of selected schizophrenia risk genes or allele, and their roles in synaptic plasticity.

Schizophrenia risk gene or allele	Mice or cell model	Deficits in synaptic plasticity	References
22q11.2DS	Df(16)1 + /− , hemizygous deletion of 23 genes in the 22q11DS-related region of mouse chromosome 16	enhanced PPF and LTP in hippocampus in mature animal	Earls et al., 2010
		
	Df(16)2 + /− , hemizygous deletion of 15 genes in the 22q11DS-related region of mouse chromosome 16	increased LTP in hippocampus in mature animal	Earls et al., 2012
		
	Df(16)5 + /− , hemizygous deletion of 6 genes in the 22q11DS-related region of mouse chromosome 16	abnormal short-term potentiation (STP) in hippocampus	Devaraju et al., 2017
		
	Df(16)A+ /-, hemizygous deletion of 27 genes in the 22q11DS-related region of mouse chromosome 16	abnormal short-term potentiation (STP) and LTP in prefrontal cortex	Diamantopoulou et al., 2017
		
	Lgdel+ /− , hemizygous deletion of 24 genes in the 22q11DS-related region of mouse chromosome 16	dysregulated network homeostatic plasticity	Amin et al., 2017
		
	DGCR8 heterozygous mutant mice	enhanced short-term synaptic depression in prefrontal cortex, increased LTP in hippocampus	Fenelon et al., 2011;Earls et al., 2012
			
NRG1	Heterozygous deletion of NRG1	impaired theta-burst LTP in hippocampus	Bjarnadottir et al., 2007
		
	bath application of NRG1 on hippocampal slices	suppressed tetanus stimulation-induced LTP in hippocampus	Chen et al., 2010
		
	conditional deletion of NRG1 in forebrain pyramidal neuron	reduced short-term potentiation (STP) and LTP in hippocampus	Agarwal et al., 2014
		
	overexpression of a cysteine-rich domain (CRD)-NRG1	reduced LTP in hippocampus	Agarwal et al., 2014
		
	prolonged treatment of cultured rat hippocampal slices with NRG1	impaired 2-AG-dependent long-term depression of inhibitory synapses	Du et al., 2013
		
	overexpression of human NRG1-IV isoform	impaired PPI, object location memory, and social interaction	Papaleo et al., 2016
DISC1	Disc1^Tm1Kara^ mice, a truncation of murine DISC1	aberrant short-term plasticity at mossy fiber–CA3 synapses in hippocampus	Kvajo et al., 2011
		
	DISC1 L100P homozygous	diminished LTP in hippocampus	Cui et al., 2016
		
	transiently disruption of DISC1’s interaction with signaling molecules Lis1 and Nudel	inhibited LTP in cortical layer 2 and 3	Greenhill et al., 2015
			
DTNBP1	Sandy mice	reduced mGluRI-dependent LTD at CA1 synapses in hippocampus	Bhardwaj et al., 2015
		
	Sandy mice	decreased inhibitory synapse numbers formed on excitatory neurons	Yuan et al., 2016
		
	Sandy mice	increase expression of NR2A in hippocampal neurons，enhanced hippocampal LTP	Tang et al., 2009
		
	Sandy mice	decreased inhibitory input to pyramidal neurons in layer V of PFC	Ji et al., 2009
		
	increased expression of dysbindin-1A in pyramidal neurons	inhibited induction of LTP in hippocampal organotypic slices	Jeans et al., 2011
		
	knockout of DTNBP1 and COMT(catechol-O-methyl transferase, another schizophrenia risk gene)	working memory deficits in a discrete paired-trial T-maze task	Papaleo et al., 2014
			
miR-137	lentiviral-mediated expression of miR-137 in dentate gyrus	impaired induction of mossy fiber LTP	Siegert et al., 2015
		
	lentiviral expressing miR-137 ‘sponge’ that causes miR-137 sequestration in dentate gyrus	enhanced induction of mossy fiber LTP	Siegert et al., 2015
		
	heterozygous conditional knockout of miR-137 in nervous system	inhibited Schaffer collateral LTP in hippocampus	Cheng et al., 2018
SATB2	Deletion of SATB2 in forebrain pyramidal neuron	deficits in maintenance of Schaffer collateral LTP	Jaitner et al., 2016

## 5 Schizophrenia risk genes play essential roles in synaptic plasticity

Earlier candidate risk gene studies have identified several schizophrenia susceptibility genes, which have been extensively investigated for their functional roles in synaptic plasticity and function. These large body of works have significantly advanced our understanding of the pathological mechanism of the disease. Moreover, accumulating data obtained from genome wide association studies (GWAS) continuously decode schizophrenia risk genes, resulting in the discovery of hundreds of disease-associated variants (Schizophrenia Working Group of the Psychiatric Genomics, C, 2014, Trubetskoy et al., 2022). Here we reviewed neurobiological studies that dissecting the roles of these schizophrenia risk genes in synaptic plasticity and function, as well as in animal behavior, hoping to provide mechanistic insight into schizophrenia pathophysiology.

### 5.1 22q11.2 microdeletion

22q11.2 microdeletion syndrome (22q11.2DS) is caused by the hemizygous deletion of a 1.5–3 Mb region on chromosome 22, which carries one of the most significant genetic risk of schizophrenia (Karayiorgou et al., 1995, Karayiorgou et al., 2010). Individuals with 22q11.2DS display a spectrum of cognitive deficits, and ∼30% of the patients develop schizophrenia in adolescence or early adulthood (Swillen et al., 2000). Several animal models have been generated to mimic 22q11.2DS, which carries hemizygous deletion of different subset of genes in the mouse chromosome 16 that are syntenic to human 22q11.2 region. These mice display core behavioral abnormalities observed in schizophrenia patients, such as impaired sensorimotor gating function and learning and memory (Devaraju et al., 2017, Fenelon et al., 2013, Paylor and Lindsay, 2006, Paylor et al., 2001, Stark et al., 2008). Moreover, abnormal or abnormally enhanced short-term and long-term plasticity in hippocampus and prefrontal cortex are frequently observed in these animal models (Devaraju et al., 2017, Diamantopoulou et al., 2017, Earls et al., 2010).

It is worth noting that studies have revealed essential effective genes contributing to the abnormal synaptic plasticity in 22q11.2DS animal models. For example, pharmacological inhibition of SERCA, a sarco(endo)plasmic reticulum Ca(2 +) ATPase, restores the enhanced LTP in hippocampus of Df(16)1^+/−^ and Df(16)2/^+/−^ mice (Earls et al., 2010, Earls et al., 2012); loss of function mutation of Mirta22, which rescues abnormal short-term potentiation (STP) and LTP in prefrontal cortex of Df(16)A^+/−^ mice (Diamantopoulou et al., 2017); hemizygous deletion of Mrpl40 contributes to STP and working memory deficits in Df(16)5^+/−^ mice, another animal model of 22q11.2DS, through dysregulation of mitochondria calcium (Devaraju et al., 2017). In addition, Dgcr8 (a gene disrupted by the 22q11.2 microdeletion) heterozygous mutant mice display smaller spines in the basal dendrites of layer 5 pyramidal neurons of the prefrontal cortex, but enhanced short-term synaptic depression (STD) in the same region (Fenelon et al., 2011). DGCR8 is essential for microRNA biogenesis. Earls et al. reported that haploinsufficiency of Dgcr8 leads to upregulation of SERCA and increased LTP, which were restored by presynaptic expression of miR-25 and miR-185 that regulates SERCA expression (Earls et al., 2012). Another 22q11.2 DS mouse model, Lgdel+ /− , display embryonic-premature alterations in the neuronal chloride cotransporters and show dysregulated network homeostatic plasticity and synchronous activity in hippocampus that might be attribute by the excitatory/inhibitory imbalance (Amin et al., 2017).

### 5.2 Neuregulin 1

Neuregulin 1 (NRG1) is a trophic factor that has an epidermal growth factor (EGF)-like domain signaling through ErbB receptor (Mei and Xiong, 2008). Genes encoding NRG1 and its predominant receptor on neuron, ErbB4 both confer risk of schizophrenia (Harrison and Weinberger, 2005). NRG1 generates many kinds of isoforms, several of which have been associated with schizophrenia-like behavior in animal model study. For instance, reduced type II neuregulin-1 in rat causes impaired visuospatial learning and working memory; whereas overexpression of type III neuregulin-1, whose expression level is increased in schizophrenia patients, in mice forebrain neurons results in decreased PPI and impaired fear memory (Olaya et al., 2018, Taylor et al., 2012). Moreover, Papaleo et al. reported that neuronal-specific overexpressing a human NRG1-IV isoform, which is increased in brain of schizophrenia individual, leads to schizophrenia-like behaviors, such as impaired PPI, object location memory, and social interaction (Papaleo et al., 2016).

In the past two decades, the major progress has been accomplished in understanding the function of NRG1 in neurotransmission and synaptic plasticity. A serial of elegant works presented that an optimized level of NRG1 signaling is critical for synaptic plasticity. Heterozygous deletion of NRG1 leads to impaired theta-burst LTP in hippocampus (Bjarnadottir et al., 2007). However, bath application of NRG1 on hippocampal slices suppressed tetanus stimulation-induced LTP (Chen et al., 2010). Consistently, Agarwal et al. reported that loss of NRG1 in cortical projection neurons disrupts LTP in hippocampus, while overexpression of the major brain isoform of NRG1, a cysteine-rich domain (CRD)-NRG1 also causes reduced LTP (Agarwal et al., 2014). GABAergic neurotransmission is involved in the regulation of NRG1 on synaptic plasticity. NRG1 deficiency or CRD-NRG1 overexpression in cortical projection neurons results in enhanced GABAergic inhibitory neurotransmission, thereby causing imbalance of excitatory-Inhibitory neurotransmission and altered LTP (Agarwal et al., 2014). Nevertheless, one possibility that NRG1 regulates LTP through an excitatory neuron-intrinsic mechanism cannot be excluded, as a recent study reported that overexpression of NRG1 in forebrain excitatory neuron in ctoNrg1 mice that mimic the upregulated level of NRG1 in schizophrenic patients, results in decreased spine density and impaired glutamatergic neurotransmission in hippocampus CA1 and PFC via activation of LIMK1 and subsequent inactivation of p-cofilin (Chen et al., 2021), which are essential for AMPAR stabilization at synapse and various forms of synaptic plasticity (Ben Zablah et al., 2021, Zhou et al., 2021).

The effect of NRG1 on inhibitory neurotransmission and LTP at hippocampus might be mediated by signaling through its receptor ErbB4 on PV-positive interneurons. ErbB4 mutation in parvalbumin (PV)-positive interneurons or acutely blocking ErbB4 enhances hippocampal LTP, suggesting that ErbB4 is a suppressor of LTP (Pitcher et al., 2008, Shamir et al., 2012). Moreover, deletion of ErbB4 in PV-positive interneurons eliminates the effect of NRG1 on increased inhibitory postsynaptic currents and suppression of LTP, while ablation of ErbB4 in pyramidal neurons has no effect (Chen et al., 2010). In addition, neuronal vesicular protein calcyon is a potent activator of NRG1 ecto-domain cleavage and overexpression of calcyon stimulates NRG1 cleavage, resulting in enhanced GABA transmission; conversely, NRG1 cleavage, ErbB4 activity and GABA transmission are decreased in calcyon null mice (Yin et al., 2015). Collectively, NRG1 activates ErbB4 in PV-positive interneurons and provokes GABA release, thereby increasing inhibitory neurotransmission and leading to diminished hippocampal LTP.

The above studies on NRG1/ErbB4 regulation of LTP mainly focused on PV-positive interneurons, however, the effect of NRG1/ErbB4 signaling on other types of interneurons remain largely elucidated. Endocannabinoids (eCBs) are implicated in the regulation of synaptic plasticity, cognition, and emotion (Castillo et al., 2012, Zanettini et al., 2011); eCBs system dysfunction is involved in schizophrenia (D'Souza et al., 2005, Giuffrida et al., 2004). The type 1 cannabinoid receptor (CB1R) is expressed in Cholecystokinin (CCK)-, but not PV-positive interneurons, suggesting an unique function of eCBs signaling in CCK-positive interneurons. Du et al. revealed that prolonged treatment of cultured rat hippocampal slices with NRG1 increased the expression of monoacylglycerol lipase (MGL), resulting in degradation of 2-arachidonolyglycerol (2-AG, one of the major eCBs) and impaired 2-AG-dependent long-term depression of inhibitory synapses (Du et al., 2013). In addition to hippocampus and PFC, amygdala is important brain region for emotional behavior and memory associated with emotion. Jiang et al. investigated the role of NRG1 in cortical glutamatergic inputs onto pyramidal neurons in basolateral nucleus of the amygdala, and found that NRG1 is also essential for multiple forms of synaptic plasticity in cortico-amygdala circuits (Jiang et al., 2013).

### 5.3 Disrupted-in-schizophrenia 1

Disrupted-in-schizophrenia 1 (DISC1) was first identified in a large Scottish family with a high loading of major mental illness (St Clair et al., 1990). The t(1; 11)(q42;q14.3) chromosomal translocation that disrupts DISC1 gene cosegregates with schizophrenia and a wide range of major mental disorders (Blackwood et al., 2001). Several mouse models carrying different DISC1 mutations have been generated to explore the function of DISC1 on schizophrenia pathology. Disc1^Tm1Kara^ mice, which contain a truncation of murine Disc1 that mimic the effects of the (1;11) translocation exhibits working memory deficits, a behavioral feature observed in individuals with schizophrenia (Kvajo et al., 2008, Kvajo et al., 2011). These mice showed aberrant short-term plasticity, but not LTP at Schaffer collateral and mossy fiber circuit in hippocampus and medial prefrontal cortex (Crabtree et al., 2017, Kvajo et al., 2008, Kvajo et al., 2011), which might be largely attributed by dysregulated PDE4, cAMP, and voltage dependent potassium channel subunit Kv1.1 (Crabtree et al., 2017, Kvajo et al., 2011). It is known that DISC1 binds directly with Pde4b and Gsk3β, two molecules involved in schizophrenia pathology. DISC1 L100P mutation affects its interaction with these two proteins, leading to deficits in working memory and object recognition memory in mice (Clapcote et al., 2007, Cui et al., 2016). Decreased synaptic transmission at DG and diminished LTP at CA1 were observed in DISC1 L100P homozygous mice (Cui et al., 2016). Moreover, Disc1 L100P mutants have defects to reorganize cortical circuitry in vivo and have impaired functional reorganization of cortical neurons in vitro in response to stimulation (Tropea et al., 2016). In addition, transiently disruption of DISC1’s interaction with signaling molecules Lis1 and Nudel during early development causes decreased AMPA/NMDA ratio and inhibited LTP in cortical layer 2 and 3 in adulthood (Greenhill et al., 2015). N-methyl-D-aspartate receptors (NMDAR) play a critical role in synaptic plasticity and cognition. It has been shown that DISC1 interacts with GluN1 subunit to regulate dendritic NMDAR motility in cultured mice neurons (Malavasi et al., 2018). Notably, DISC1 might interact with other schizophrenia susceptibility factors. it was shown that NRG1 and DISC1 link directly into a common pathway mediated by Erb (ErbB2 and ErbB3) receptors and P13K/AKT1 signaling (Seshadri et al., 2010). These findings suggest that NRG1, ErbBs and DISC1 synergistically regulates neurodevelopment to contribute to schizophrenia pathology.

### 5.4 Dystrobrevin binding protein 1

DTNBP1 (dystrobrevin binding protein 1) encodes a coiled-coil-containing protein commonly called dysbindin-1, one of the subunits of biogenesis of lysosome-related organelles complex 1 (BLOC-1) involved in protein trafficking. DTNBP1 is associated with increased risk of schizophrenia and its mRNA and protein level are reduced in postmortem brain from schizophrenia patient (Straub et al., 2002, Talbot et al., 2004, Weickert et al., 2008). Majority of the functional investigations on DTNBP1 were performed on Sandy (Sdy) mice that have a nature loss of function mutation in DTNBP1 gene resulting in no dysbindin-1 protein expression. Sdy mice showed an overactivation of dopamine D2 receptor (D2R), and the subsequent spine deficiency, dysconnectivity in the entorhinal-hippocampal circuit and impaired working memory (Jia et al., 2013). Moreover, loss of dysbindin-1 in Sdy mice leads to impaired group 1 metabotropic glutamate receptor (mGluRI)-extracellular signal regulated kinase 1/2 (ERK1/2) signaling and reduced mGluRI-dependent LTD at CA1 synapses (Bhardwaj et al., 2015). Notably, enhancing mGluR5 transmission with CDPPB ameliorates deficits in short-term novel object recognition memory in Sdy mice (Bhardwaj et al., 2015). Sdy mice also displayed increased surface expression of NR2A in hippocampal neurons and enhanced hippocampal LTP, but intact basal synaptic transmission, presynaptic properties, and LTD (Tang et al., 2009). In addition, dysbindin also contributes to cortical interneuron development and network activity. Loss of dysbindin-1 resulted in increased D2 signaling and decrease in the excitability of fast-spiking GABAergic interneurons in layer V of PFC (Ji et al., 2009); dysbindin-1 mutation reduced the exocytosis of BDNF from cortical excitatory neurons, decreasing inhibitory synapse numbers formed on excitatory neurons (Yuan et al., 2016). Double knockout of DTNBP1 and COMT (catechol-O-methyl transferase, another schizophrenia risk gene) causes working memory deficits in a discrete paired-trial T-maze task, which is highly associated with dopamine function in PFC (Papaleo et al., 2014). However, increased expression of dysbindin-1A in pyramidal neurons inhibits NMDAR function and blocks induction of LTP in hippocampal organotypic slices (Jeans et al., 2011).

### 5.5 miR-137

In addition to risk alleles locate at coding region, non-coding genes are also implicated in schizophrenia, such as microRNAs that play important roles in regulating gene expression at posttranscriptional level. miR-137 gain-of-function in vivo with lentiviral-mediated expression of miR-137 in dentate gyrus resulted in changed synaptic vesicle pool distribution, impaired induction of mossy fiber-LTP and deficits in hippocampus-dependently learning and memory (Siegert et al., 2015); while lentiviral expressing miR-137 ‘sponge’ that sequester miR-137 reverses the effect of miR-137 gain of function on LTP and behavior. Pre-synaptic mechanism might underly the regulation of miR-137 on mossy fiber-LTP and memory, as overexpression of miR-137 leads to downregulation of presynaptic targets genes including Complexin-1 (Cplx1) and Synaptotagmin-1 (Syt1) and impaired vesicle release (Siegert et al., 2015). Moreover, Chen et al. observed that complete loss of miR-137 in mice leads to postnatal lethality and miR-137 heterozygous conditional knockout mice exhibit inhibited Schaffer collateral LTP and impaired learning and social behavior, which is mediated by miR-137 target phosphodiesterase 10a (Pde10a) (Cheng et al., 2018).

### 5.6 SATB2

SATB2 is a schizophrenia risk gene encodes a DNA-binding protein (Schizophrenia Working Group of the Psychiatric Genomics, 2014). SATB2 is highly enriched in pyramidal neurons in cerebral cortex and the hippocampal CA1, two brain regions significant for memory formation (Jaitner et al., 2016). Deletion of SATB2 in forebrain of adult mice causes deficits in maintenance of Schaffer collateral LTP and impairs long-term fear and object discrimination memory (Jaitner et al., 2016). The underlying mechanism of SATB2 in synaptic plasticity remains complicated, as SATB2 is involved in a complex gene regulation network. SATB2 regulates a large amount of miRNAs in the hippocampal, many of which are involved in synaptic plasticity and memory formation (Jaitner et al., 2016). Meanwhile, interaction between SATB2 and the inner nuclear membrane protein LEMD2 alters expression of numerous genes that are related to schizophrenia etiology in pyramidal neurons (Feurle et al., 2021). Another gene set enrichment analysis showed that genes functionally related to or targeted by SATB2 are enriched for genes associated with schizophrenia (Whitton et al., 2018).

Growing evidence has shown that many other genes associated both with schizophrenia and synaptic plasticity, like SNAP25 (Baca et al., 2013), GRIN3B (Hornig et al., 2017), NRN1 (Fatjo-Vilas et al., 2016), NRGN (Hwang et al., 2021) and so on. However, there is still a very tough and long way to dissect the role of risk genes on synaptic plasticity and unveil the pathogenesis of schizophrenia and other kinds of mental disorder.

## 6 Environmental factors and synaptic plasticity disturbance

Many environmental factors, including prenatal immune activation, contribute to the onset of schizophrenia. The interference of early life infection on brain development is consistent with the neurodevelopmental hypothesis of schizophrenia, that is, abnormal early brain development is one of the causes of schizophrenia. Viral infections of central nervous system during perinatal (Brown, 2006) and childhood (Khandaker et al., 2012) increase the risk of mental illness in adulthood. A Finnish study showed that children with central nervous system infections were five times more likely to develop schizophrenia than the control group (Jones, 1998). Injection with viral analogue PolyI:C on gestation day 9 of pregnancy caused global DNA hypomethylation and enrichment of differentially methylated genes related to synaptic plasticity in mice offspring (Basil et al., 2018), implying that maternal immune activation affects synaptic plasticity in adult offspring.

MAM (Methylazoxymethanol) is an anti-mitotic agent that methylates DNA. It has been reported that MAM-exposure in embryonic day 17 (E17) contributes to schizophrenia-like behavior both in mice and rats (Huo et al., 2018, Moore et al., 2006). Intriguingly, MAM treatment in E17 mice leads to gender specific deficits in PPI and social recognition, as well as many differentially expressed genes relevant to synaptic plasticity (Huo et al., 2018). Moreover, disruption of one-carbon metabolism by increasing methionine daily intake in pregnant mice during the last week of gestation causes schizophrenia-like behavior including impaired social recognition, sensorimotor gating function, and working memory in offspring (Alachkar et al., 2018). However, electrophysiological examination of different forms synaptic plasticity in detail in these animal models are needed to characterize how schizophrenia-related environmental changes affect synaptic plasticity.

## 7 Conclusion

Schizophrenia is a deliberating mental illness affecting 1% human populations. Dysregulated synaptic transmission and plasticity have been implicated in the pathogenesis of the illness, although the underlying mechanism has yet determined. Synaptic plasticity plays a very significant role in normal brain function and development including the formation of correct circuit connection. In this review article, firstly, we discussed molecular mechanisms of various types of synaptic plasticity. LTP is important for memory formation and preservation and LTD is necessary for the reconstruction of synapse connection for LTP. Meanwhile, homeostatic plasticity keeps neuron activity in a moderate range. Secondly, we summarized the susceptibility gene and related animal models of schizophrenia, with the emphasis on risk genes that are known to modulate synaptic plasticity. In addition to genetic factors, environmental factors like viral infection, autoimmune disease or social isolation during childhood also contribute to the onset of schizophrenia. Thirdly, we proposed that environmental alterations might also lead to schizophrenia-like behavior and deficits in synaptic plasticity. As we reviewed here, each kind of plasticity plays a very important role in maintaining the normal function of the nervous system. Any imbalance in plasticity will pose a risk of mental disorder including schizophrenia. Given an rapidly expanded list of schizophrenia risk genes characterized by GWAS, functional studies to further dissect the roles of these genes in regulating synaptic plasticity and function are urgently needed to uncover the neuropathological mechanism of schizophrenia and develop new targets for clinical interventions.
